# The Impact of Atorvastatin on Intraprostatic Biomarkers – Prognostic Value of 3LS-score – Follow-up of ESTO1-Trial

**DOI:** 10.1016/j.neo.2025.101132

**Published:** 2025-02-06

**Authors:** Eemil Lehtonen, Maiju Vertanen, Heimo Syvälä, Teemu Tolonen, Seppo Auriola, Teuvo Tammela, Aino Siltari, Teemu Murtola

**Affiliations:** a Faculty of Medicine and Health Technology, Tampere University, Tampere, Finland; b Tampere University, Faculty of Medicine and Health Technology, Tampere, Finland; Department of Pathology, FimLab Laboratories, Tampere, Finland; c School of Pharmacy, University of Eastern Finland, Kuopio, Finland; d Tampere University, Faculty of Medicine and Health Technology, Tampere, Finland; TAYS Cancer Center, Department of Urology, Tampere, Finland; e Faculty of Medicine and Health Technology, Tampere University, Tampere, Finland; Department of Pharmacology, Faculty of Medicine, University of Helsinki, Helsinki, Finland; f Faculty of Medicine and Health Technology, Tampere University, Tampere, Finland; TAYS Cancer Center, Department of Urology, Tampere, Finland

**Keywords:** Prostate cancer, Statins, Lipidomics, Three-lipid signature, Ki67, Biochemical recurrence, Cardiovascular risk

## Abstract

**Background:**

Prostate cancer (PCa) remains a global health burden, with limited reliable biomarkers beyond prostate-specific antigen (PSA). Statins have been associated with survival benefits in advanced Pca, potentially by modulating cholesterol metabolism and tumor biology. However, the causal mechanisms are not well understood. A distinct three-lipid signature (3LS) has previously been proposed as a prognostic biomarker for PCa.

**Objective:**

This study investigates the effects of atorvastatin intervention on PCa tissue markers, long-term clinical outcomes, and the prognostic value of the 3LS derived from prostate tissue lipidome.

**Methods:**

The ESTO1 trial randomized 158 statin-naïve PCa patients to receive high-dose atorvastatin (80 mg daily) or placebo before prostatectomy. Long term outcomes were assessed for 102 patients through medical records review. Prostate tissue samples were pathologically characterized, and lipidome quantified. Cox regression models were used to analyse clinical outcomes between the groups. The 3LS score was calculated by identifying the constituent lipids from the prostate lipidome.

**Findings:**

Higher intraprostatic atorvastatin lactone concentrations were associated with reduced Ki67 expression and PSA levels. After a median follow-up of seven years, no significant differences were observed in biochemical recurrence, overall mortality, or initiation of hormonal therapy. However, the atorvastatin arm had a lower risk of major acute cardiovascular events (HR 0.11, 95% CI 0.01–1.01). The intraprostatic 3LS correlated with higher baseline tumor aggressiveness but did not predict subsequent outcomes.

**Conclusion:**

Higher atorvastatin lactone concentrations in the prostate tissue were linked to improved pathological variables. Pre-surgery statin intervention reduced MACE risk but no impact on other clinical outcomes was observed. The 3LS from prostate tissue does not seem to be prognostic marker in localized Pca.

## Research in context

Evidence before this study

- Statins are one of the most widely prescribed drug class globally. In addition to their well-established cholesterol-lowering effects, statins have demonstrated confirmed and potential anti-cancer properties through cholesterol-dependent and other mechanisms. Epidemiological studies have associated statin use with significant survival benefits in advanced prostate cancer. However, only few window-of-opportunity randomized trials have evaluated statin therapy in prostate cancer patients, with inconclusive results.

Added value of this study

- Statin therapy administered before scheduled prostatectomy resulted in a reduced risk of major acute cardiovascular events during long term follow-up. Intraprostatic atorvastatin concentrations correlated with improved pathological markers in prostate tissue.

Implications of all the available evidence

- Future prospective studies should focus on statin therapy using higher doses and longer exposure, particularly in patients with more advanced prostate cancer. Effects of lactone form and active hydroxy acid form of statins should be assessed separately.

## Introduction

Prostate cancer (PCa) is one of the most prevalent malignancies among men worldwide and remains a leading cause of cancer-related mortality in developed countries [1,2]. PCa presents with heterogeneous prognoses: most diagnosed PCas are benign and/or curatively treatable. However, approximately 10–15% of cases progress to incurable metastatic disease associated with high mortality rates.

Well-established prognostic factors for PCa progression include Gleason score, disease stage, and serum prostate-specific antigen (PSA) levels at diagnosis [3]. Currently, PSA is the sole biomarker routinely used in clinical use for PCa diagnosis. However, its utility as a screening tool is controversial due to potential overdiagnosis and overtreatment, which may outweigh its benefits.

Risk factors of PCa are not yet completely understood. Known risk factors include age, African American ancestry, and family history of PCa. Modifiable factors, such as serum cholesterol, have also been implicated in PCa development. effect on PCa development. Epidemiological evidence suggest that elevated blood cholesterol levels may be a risk factor for PCa cancer, particularly for advanced or metastatic cancer [4,5].

Cholesterol plays a critical role in cellular functions, including maintaining cell membrane structure, enabling cell signaling, and serving as a precursor for steroid biosynthesis [6]. Statins, which are commonly used for the primary and secondary prevention of cardiovascular, inhibit HMG-CoA reductase, a rate-limiting enzyme of the cholesterol-producing mevalonate pathway [7]. Additionally, statins affect protein isoprenylation, a critical process for the proper functioning of small G-proteins. These proteins play important roles in cell proliferation, metastasis, and apoptosis [7]. This is essential as uncontrolled cell division and the inability to undergo apoptosis are well-known hallmarks of cancer [8]. Our previous research demonstrated that low-density lipoprotein (LDL) cholesterol accelerates PCa cell growth, and that cholesterol synthesis is significantly more active in cancer cells than in normal cells [9]. Consequently, statins could have the potential to affect PCa development through altering cell signalling and cell proliferation, both by reducing cholesterol levels and affecting the small G-proteins [10]. Additionally, reducing cholesterol levels decrease intraprostatic androgen levels that are critical for PCa growth [11].

In epidemiological studies, statins use has been associated with a 20% reduction in the risk of developing advanced PCa [12]. However, findings regarding overall PCa risk have been contradictory. Additionally, statin use has been linked to a 20-30% reduction in PCa-spesific mortality rate compared to non-users [11,13,14]. The association is particularly strong in men on androgen deprivation therapy [15,16]. The impact of statin treatment on PCa mortality appears to depend on serum cholesterol levels, with a reduction in mortality observed among statin users only when serum cholesterol levels are lowered. Furthermore, statins appear to enhance the efficacy of drugs targeting androgen signalling, such as abiraterone and enzalutamide [[17], [18], [19]].

To date, only three randomized controlled trials have evaluated the effects of statin interventions in Pca patients [[20], [21], [22]]. In the ESTO1 study, our research group demonstrated that neoadjuvant atorvastatin before prostatectomy reduced cancer tissue proliferation activity and serum PSA levels compared to placebo. Additionally, atorvastatin treatment affected lipidome and steroid hormones both in the serum and prostate tissue [23,24]. Atorvastatin was also detectable in PCa tissue, supporting the hypothesis of direct statin effects on prostate tissue [25]. Similar results have been reported with fluvastatin [22]. However, low-dose atorvastatin did not reduce recurrence risk in high-risk patients after prostatectomy [21].

If the possible survival benefit associated with statins is to be understood better, further research is needed to characterize systemic and local lipidome changes and assess their diagnostic and predictive value in PCa development and progression

Previously described by Lin et al. [26], a lipid signature comprising ceramide, sphingomyelin, and phosphatidylcholine has been associated with adverse outcomes in PCa, including metastatic relapse, earlier testosterone suppression failure, and shorter survival in advanced disease [27,26]. To our knowledge this signature and its possible prognostic value has not been previously studied from lipid concentrations measured from intraprostatic samples.

This study aims to evaluate correlations between the intraprostatic 3LS and atorvastatin concentrations with Pca tissue markers, including Ki67, inflammation scores, and PSA. Additionally, we will perform follow-up analysis of the ESTO1 trial to assess the possible long-term effects of atorvastatin intervention on biochemical relapse and survival outcomes following primary curative treatment with radical prostatectomy. We will also examine the prognostic role of the intraprostatic 3LS score in relation to survival outcomes in localized PCa.

## Materials & methods

### Study cohort

The primary results of ESTO1 study have been published previously [20,24]. The study explored the effects of high-dose atorvastatin on prostate tissue before prostatectomy (ESTO1-trial, EudraCT 2011-005438-20). A total of 158 (160) statin-naïve PCa patients scheduled for radical prostatectomy were randomized in a 1:1 ratio to use either 80 mg atorvastatin daily or a placebo in a double-blind fashion from recruitment until surgery. No minimum duration for the intervention was set, with a median duration of 27 days (IQR 10-114) in the atorvastatin group and 27 days (4-76) in the placebo group. Prostatectomies and subsequent follow-up visits were conducted according to standard clinical practice. The design and sample collection protocols for the ESTO1 trial have been described in detail elsewhere [[23], [24], [25],^28^,^29^]

Blood samples for serum PSA measurement were collected from participants before the intervention and prior to surgery. Atorvastatin was orally administered as the calcium salt of the standard active hydroxy acid form. In vivo it is converted to, and is in equilibrium with, its lactone form. The key active metabolites of the lactone form account for majority of the HMG-CoA reductase inhibition with minor contribution from the atorvastatin hydroxy acid. Both atorvastatin and atorvastatin lactone concentrations were quantified from pre-surgery blood samples. Fresh prostate tissue samples were collected during the surgery, and mass spectrometer was used to determine atorvastatin and atorvastatin lactone levels in the tissue.

The whole prostate was embedded in paraffin blocks and immunohistochemical staining was performed using a modified LabVision Autostainer with antigen retrieval at 121°C for 2 minutes in Tris-EDTA buffer (pH 9). Sections were counterstained with hematoxylin and scanned as high-resolution virtual slides [30]. Intraprostatic inflammation score and expression of tumor proliferation marker Ki67 were determined by study pathologists from tissue slides cut from the blocks. The study pathologists determined both the highest and median levels of Ki67 from the immunohistochemical slides. Ki-67 labeling index was analyzed from this macro slices, blinded to treatment allocation, using ImmunoRatio 1.0c software on three macroscopic hotspots with the highest Ki-67 labeling [31]. Quantifications were reviewed by another pathologist and differences discussed until consensus was reached. The highest level represents the peak expression observed, while the median level represents the median of expression across the patient's sample. Inflammation scores were assigned based on the international consensus criteria [32].

For the first part of the study, which utilizes the post and pre-surgery tissue and blood samples, the analyses were completed on the full cohort (n=158). This included the further evaluation of how atorvastatin and atorvastatin lactone concentrations (non-measurable vs. measurable, median or below, and above median) in both serum and prostate tissue impacted on Ki67 expression, inflammation score, variations in PSA levels, and 3LS score. Atorvastatin and atorvastatin lactone concentrations were available for 57 of the 80 men who completed the atorvastatin intervention. Of these 57, atorvastatin concentration in the prostate was non-measurable in 15 subjects and atorvastatin lactone concentration in 4 subjects. Atorvastatin and atorvastatin lactone concentrations in plasma were measurable in all the patients. For further analysis, we combined the atorvastatin and atorvastatin lactone concentration and created one variable of this combination.

The second phase involved a follow-up of the ESTO1 cohort to assess long-term outcomes, requiring no active participation from the patients. Follow-up data were collected by gathering information from electrical medical records, including information on PSA relapse, major acute cardiovascular events (ischemic stroke and myocardial infarction), initiation of hormonal therapy, and mortality. While mortality data were available for all patients, information on biochemical relapse, cardiovascular events and ADT initiations was complete only for patients residing in the Pirkanmaa region (n = 102). The cause of death could only be assessed if it was noted on the electrical medical records. Analyses were completed for both the follow-up cohort and the full cohort, acknowledging that not all data was available for the full cohort.

### Lipidome measurement

A fresh cut from macroscopically cancer-free tissue was collected during surgery and stored in liquid nitrogen for subsequent lipidome profiling. The intraprostatic lipidome was measured using liquid chromatography high-resolution mass spectrometry (LC–MS/MS), as previously described by Raittinen et al. [24]. The intraprostatic lipidome was determined for 144 patients and the three lipids of interest were identified from the sample. The mass spectrometry signals were normalized and transformed into logarithm scale for statistical analyses. The distinct three-lipid signature was calculated as described by Lin [26] from the prostate tissue concentration s of ceramide (d18:1/24:1), sphingomyelin (d18:2/16:0), and phosphatidylcholine (16:0/16:0), using formula y=3.1319*c1+2.1724*c2+1.8593*c3 and p=e^y^/(1+e^y^) where c1, c2, and c3 are the concentrations of ceramide, sphingomyelin, and phosphatidylcholine respectively. Patient had the 3LS signature if p≥0.5.

### Statistical analysis

Comparisons between atorvastatin and atorvastatin lactone concentrations and study endpoints were conducted using the Mann-Whitney U test for continuous variables and Pearson's chi-squared test for categorical variables. Continuous variables included Ki67 expression (highest and median), inflammation scores, baseline PSA, pre-surgery PSA, and PSA changes. The 3LS score was analysed as a binary categorical variable (positive or negative).

For the follow-up cohort, associations of statin intervention and clinical outcomes were analyzed using multiple Cox proportional hazard models, with either biochemical recurrence, major acute cardiovascular outcome, hormonal therapy inhiation or mortality as dependent variable. Hazard ratios (HRs) are presented for both unadjusted models, where the treatment arm was the sole predictor, and adjusted models, which included age at recruitment, pathological T-stage, and baseline PSA as additional covariates.

For individuals in the follow-up cohort who had their intraprostatic lipidome assessed, we performed similar Cox model analyses to evaluate the prognostic association of 3LS status. Additionally, to address calibration and cut point challenges when applying the serum-derived 3LS formula to intraprostatic measurements, we developed a univariable Cox regression model with the p-term of the 3LS formula as a continuous variable, modelled using restricted cubic splines to allow for a flexible relationship with biochemical recurrence survival risk.

Ethical approval for the follow-up study was applied from the Pirkanmaa wellbeing county's ethical board (R24012l). All statistical analyses were done with R software version 4.3.2 or IBM SPSS version 28.0.1.0.

## Results

### Population characteristics

**Fig. 1:**
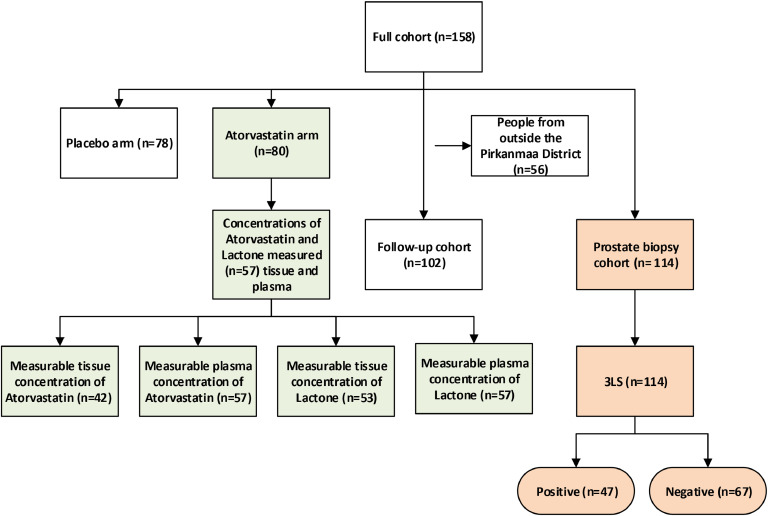
Study flow-chart of the follow-up of patients with prostate cancer treated with radical prostatectomy and participating in ESTO1 clinical trial. legend: Participants in the ESTO1 trial were randomized to use a daily dose of either 80 mg atorvastatin or a placebo from the day of recruitment until surgery. Long term follow-up information was not reliably available for patients residing outside the Pirkanmaa district. 3LS is a distinct lipid signature calculated in this study from the in from the intraprostatic concentrations of three lipids that have been previously shown to have prognostic value in prostate cancer as measured from serum.

The original ESTO1 trial recruited 158 PCa patients between September 2012 and January 2016. Out of these, 102 patients had complete follow-up data available through November 30, 2022 (Fig. 1). Table 1 summarizes the characteristics of the full study population, the follow-up cohort, and subgroups with and without 3LS present. Population characteristics were comparable across the different cohorts (Table 1). The median follow-up time was 94.5 months (IQR 87.0–106.5) for full cohort and 96.0 months (86.5–108.0) for the follow-up cohort. The median age of patients was 64 years (58–68) for both the full cohort and follow-up cohort. Median body mass index (BMI) values in the groups were correspondingly 26.5 (24.6–29.2) and 26.8 (24.7-29.6). Current smokers comprised 13-18% of the patients in the cohorts.

**Table 1:** Clinical characteristics of the study population of 158 prostate cancer patients treated with radical prostatectomy and participating in ESTO1 clinical trial

Characteristic	Full cohort**	Follow-up cohort	3LS present	3LS absent
N of men:	158	102	48	66
Atorvastatin arm (%)	78 (49.4)	59 (57.8)	23 (47.9)	31 (47)
Placebo arm (%)	80 (50.6)	43 (42.2)	25 (52.1)	35 (53)
Follow-up (months); median (IQR)	94.5 (87.0–106.5)	96.0 (86.5–108.0)	94.4 (87.5-106.3)	94.2 (86.7-109.3)
Age at baseline; median (IQR)	64.0 (58.0-68.0)	64 (58.0-68.0)	65.0 (61.8-68.0)	64.0 (58.0-68.0)
Smoking				
Current (1) (%)	24 (15.2)	18 (17.6)	7 (14.6)	11 (16.7)
Former (3) (%)	3 (1.9)	3 (2.9)	0 (0)	3 (4.5)
Never (0) (%)	123 (77,8)	77 (75.5)	40 (83.3)	49 (74.2)
BMI; median (IQR)	26.45 (24.56-29.22)	26.78 (24.68-29.57)	26.7 (24.4-29.2)	26.6 (24.9-29.7)
N of men with atorvastatin concentration in the prostate measured (%*)	57 (73.1)	41 (69.5)	18	25
N of men with atorvastatin concentration in the plasma measured (%*)	57 (73.1)	41 (69.5)	18	18
N of men with lactone concentration in the prostate measured (%*)	57 (73.1)	41 (69.5)	18	25
N of men with atorvastatin lactone concentration in the plasma measured (%*)	57 (73.1)	41 (69.5)	18	25
Median (IQR) atorvastatin concentration (ng/g) in the prostate	3.31 (0.00-18.96)	2.58 (0.00-15.11)	2.1 (0.4-6.2)	13.0 (0.6-22.6)
Median (IQR) atorvastatin concentration (ng/ml) in the plasma	1.69 (0.71–21.56)	1.63 (0.71–19.97)	1.6 (0.5-18.7)	1.6 (0.8-27.0)
Median (IQR) atorvastatin lactone concentration(ng/g) in the prostate	1.66 (0.39–6.87)	1.15 (0.41–5.57)	0.7 (0.3-4.1)	3.7 (1.0-10.7)
Median (IQR) atorvastatin lactone concentration (ng/ml) in the plasma	1.81 (0.72-22.25)	1.73 (0.7-17.79)	1.61 (0.62-24.91)	2.15 (0.81-22.99)
ISUP Gleason score at prostatectomy:				
1 n (%)	34 (21.5)	22 (21.6)	5 (10.4)	19 (28.8)
2 n (%)	89 (56.3)	59 (57.8)	30 (62.5)	36 (54.5)
3 n (%)	18 (11.4)	11 (10.8)	6 (12.5)	7 (10.6)
4 n (%)	6 (3.8)	5 (4.9)	2 (4.2)	2 (3.0)
5 n (%)	9 (5.7)	5 (4.9)	5 (10.4)	2 (3.0)
Pathological T-stage:				
T1-2 n (%)	86 (55.1)	59 (57.8)	25 (52.1)	36 (54.5)
T3-4 n (%)	70 (44.9)	43 (42.2)	22 (45.8)	29 (43.9)
Pathological N-stage:				
N1 n (%)	5 (21.7)	2 (14.3)	5 (71.4)	7 (70.0)
N0/x n (%)	18 (78.3)	12 (85.7)	2 (28.6)	3 (30.0)
PSA at baseline; median (IQR)	7.8 (5.5–11.0)	7.5 (5.6–10.2)	8.5 (5.5-12.3)	7.5 (5.5-9.8)
Biochemical recurrence; n (%)	35 (22.2)	26 (25.5)	11 (31)	16 (37)
Median (IQR) time to biochemical recurrence (months)	34 (12-49)	36 (15-51)	35.2 (12-55)	33.6 (14-50)
PSA at the time of relapse; median (IQR)	0.14 (0.05–0.30)	0.14 (0.05–0.30)	0.08 (0.06-0.30)	0.07 (0.05-0.19)
Deaths; n (%)	10 (6.3)	7 (6.9)	5 (10.4)	3 (4.5)
Other cause of death, n (%)	3 (1.9)	2 (2.0)	2 (4.2)	1 (1.5)
Unknown cause of death, n (%)	7 (4.4)	5 (4.9)	3 (6.3)	2 (3.0)
Major acute cardiovascular event; n(%)	6 (3.80)	6 (5.9)	3 (6.3)	2 (3.0)
Hormonal treatment; n(%)	12 (11.8)	10 (9.8)	4 (9.5)	3 (4.8)
Inflammation score	9.0 (7.0-12.0)	10.0 (8.0-12.0)	10.0 (7.8-12.0)	9.0 (7.0-11.0)
Median Ki67; median (IQR)	1.8 (1.2-2.775)	1.8 (1.1-2.65)	2.2 (1.4-3.1)	1.7 (1.1-2.5)
Highest Ki67; median (IQR)	2.35 (1.5-3.7)	2.2 (1.43-3.35)	2.6 (1.9-3.7)	2.1 (1.4-3.0)

* Percentage of men in the atorvastatin arm of ESTO1 trial

** These data are partly incomplete because information about all those outside Tays is not obtained

### Impact of Atorvastatin and Atorvastatin Lactone Concentrations on Prostate Tissue Markers

Patients with measurable intraprostatic concentrations of atorvastatin exhibited lower median Ki67 values compared to those with non-measurable concentrations, although the difference did not reach statistical significance (p=0.195) (Table 2). Median PSA levels at baseline were lower in the non-measurable atorvastatin group compared to the measurable group (p=0.014). However, pre-surgery PSA levels were not statistically significantly different between the groups (p=0.085). Median decrease of PSA was higher in the measurable group (p=0.237), also not statistically significantly. No association with intraprostatic inflammation score was observed.

**Table 2:** Tissue concentration of atorvastatin of patients with prostate cancer treated with radical prostatectomy and participating in ESTO1 clinical trial (p-values when comparing to non-measurable)

	Tissue concentration of atorvastatin						
	*Non-measurable (n=15)*	*Measurable (n=42)*	*p-value*	*Median or below (n=14)*	*p-value*	*Above median (n=28)*	*p-value*
Highest Ki67; median (iqr)	3.2 (1.7-4.4)	2.3 (1.25-3.55)	0.267	2.14 (1.175-3.625)	0.213	2.3 (1.8-3.5)	0.408
Median Ki67; median (iqr)	2.5 (1.4-3.6)	1.8 (1.1-2.7)	0.195	1.2 (0.9-3.1)	0.128	1.8 (1.275-2.625)	0.357
Intraprostatic inflammation score; median (iqr)	10 (9-12)	9 (8-12)	0.412	9.5 (8-12)	0.877	8 (7-11)	0.285
PSA baseline; median (iqr)	5.6 (4.4-7.9)	8.7 (5.85-13.625)	0.014	8.7 (4.625-14.2)	0.144	8.7 (6.15-13.475)	0.01
PSA before surgery; median (iqr)	6.25 (4.1-7.975)	7.25 (5.45-12.875)	0.085	10.1 (5.2-12)	0.323	9 (4.95-9.75)	0.064
PSA change; median (iqr)	-0.4 (-1.225-1.125)	-0.65 (-2.175-0.15)	0.237	-0.450 (-1.375-0.425)	0.505	-0.9 (-2.625-0.1)	0.2
3 ls score neg/pos; n (%)	4 (50%)/ 4 (50%)	21 (60%)/14 (40%)	0.605	5 (41.7%)/ 7 (58,3%)	0.248	16 (69,6%)/ 7 (30,4%)	0.248
	Tissue concentration of atorvastatin lactone						
	*Non-measurable (n=4)*	*Measurable (n=53)*	*p-value*	*Median or below (n=25)*	*p-value*	*Above median (n=28)*	*p-value*
Highest Ki67; median (iqr)	3.8 (3.625-8.325)	2.25 (1.425-3.65)	0.032	2.3 (1.4-3.85)	0.054	2.1 (1.4-3.5)	0.029
Median Ki67; median (iqr)	3.45 (2.7-5.775)	1.75 (1.175-2.8)	0.024	1.95 (1.125-3.075)	0.057	1.75 (1.175-2.375)	0.016
Intraprostatic inflammation score; median (iqr)	9 (5-13.75)	9 (8-12)	0.911	10 (8-12)	0.773	9 (8-11)	0.953
PSA baseline; median (iqr)	5.67 (3.11-13.375)	8.4 (5.55-12.05)	0.365	5.8 (5-10.25)	0.658	9.25 (6.675-13.475)	0.21
PSA before surgery; median (iqr)	7.5 (3.65-14.35)	7.15 (5.1-10.95)	0.886	6.6 (5.3-9.45)	0.554	9.4 (4.95-10)	0.798
PSA change; median (iqr)	1.05 (0.15-2.145)	-0.6 (-1.975-0.075)	0.014	-0.5 (-1.3-(-0.1))	0.014	-1.25 (-2.625-0.25)	0.024
3 ls score neg/pos; n (%)	2 (66,7%)/ 1 (33,3%)	23 (57,5%)/ 17 (42,5%)	0.756	8 (47,1%)/ 9 (52,9%)	0.491	15 (65,2%)/ 8 (34,8%)	0.491
	Tissue concentration of atorvastatin and atorvastatin lactone combined						
	*Non-measurable (n=2)*	*Measurable (n=55)*	*p-value*	*Median or below (n=26)*	*p-value*	*Above median (n=29)*	*p-value*
Highest Ki67; median (iqr)	3.8 (3.7-.)	2.3 (1.475-3.725)	0.208	2.25 (1.45-4.1)	0.305	2.3 (1.5-3.475)	0.157
Median Ki67; median (iqr)	3.05 (2.5-.)	1.8 (1.2-2.875)	0.199	1.7 (1.15-3.3)	0.308	1.8 (1.2-2.6)	0.143
Intraprostatic inflammation score; median (iqr)	7 (4-.)	9 (8-12)	0.398	10 (8-12)	0.32	8.5 (7.25-11)	0.502
PSA baseline; median (iqr)	5.67 (4.34-.)	8.4 (5.5-12.5)	0.278	6.65 (4.85-10.7)	0.475	8.7 (6.3-13.45)	0.171
PSA before surgery; median (iqr)	7.5 (6.8-.)	7.15 (5.1-11.575)	0.808	6.2 (4-10.75)	0.643	7.3 (5.75-11.75)	0.968
PSA change; median (iqr)	1.83 (1.2-.)	-0.55 (-1.725-0.1)	0.032	-0.4 (-1.3-0.15)	0.029	-1.1 (-2.55-0.1)	0.044
3 ls score neg/pos; n (%)	0 (0%)/1 (100%)	25 (59,5%)/17 (40,5%)	0.233	9 (50%)/9 (50%)	0.273	16 (66,7%)/8 (33,3%)	0.273

Measurable tissue concentrations of atorvastatin lactone were associated with a reduction in both median Ki67 (p=0.024) and highest Ki67 values (p=0.032). PSA levels also decreased more in the measurable atorvastatin lactone group compared to the non-measurable group (p=0.014). Similar effects were observed for the combined variable representing both atorvastatin and atorvastatin lactone concentrations (p=0.032). No significant associations were detected between higher atorvastatin lactone concentrations and intraprostatic inflammation scores. It is worth noting that the number of patients with non-measurable atorvastatin lactone concentrations was only four (Table 2).

As expected, atorvastatin and atorvastatin lactone concentrations were detectable in plasma for all participants in the intervention group (Table 3). However, higher plasma concentrations of either compound were not significantly associated with lower Ki67 values, PSA levels, or intraprostatic inflammation scores.

**Table 3:** Tissue concentration of atorvastatin lactone of patients with prostate cancer treated with radical prostatectomy and participating in ESTO1 clinical trial (p-values when comparing to non-measurable)

	Plasma concentration of atorvastatin			
	Non-measurable (n=0)	Median or below (n=28)	Above median (n=29)	p-value
Highest Ki67; median (iqr)	-	2.25 (1.55-3.925)	2.35 (1.325-3.725)	0.889
Median Ki67; median (iqr)	-	1.8 (1.2-3.225)	1.9 (1.1-2.575)	0.749
Intraprostatic inflammation score; median (iqr)	-	9 (8-12)	9 (8-12)	0.779
PSA baseline; median (iqr)	-	8.3 (5.525-14.1)	8.4 (5.2-10.25)	0.363
PSA before surgery; median (iqr)	-	8.2 (5.1-13.6)	6.2 (5-8)	0.103
PSA change; median (iqr)	-	-0.4 (-1.5-0.5)	-0.7 (-1.85-0.05)	0.56
3 ls score neg/pos; n (%)		13 (59,1%)/ 9 (40,9%)	12 (57,1%)/ 9 (42,9%)	0.897
	Plasma concentration of atorvastatin lactone			
	*Non-measurable (n=0)*	*Median or below (n=28)*	*Above median (n=29)*	*p-value*
Highest Ki67; median (iqr)	-	2.3 (1.425-3.925)	2.3 (1.725-3.725)	0.980
Median Ki67; median (iqr)	-	2.1 (1.2-3.225)	1.75 (1.075-2.575)	0.396
Intraprostatic inflammation score; median (iqr)	-	9 (7.25-12)	10 (8-12)	0.754
PSA baseline; median (iqr)	-	9.65 (5.125-14.1)	7.7 (5.5-9.95)	0.247
PSA before surgery; median (iqr)	-	8.2 (5.1-13.6)	6.8 (5-8)	0.154
PSA change; median (iqr)	-	-0.4 (-2.4-0.5)	-0.6 (-1.6-0.05)	0.902
3 ls score neg/pos; n (%)		12 (57,1%)/9 (42,9%)	13 (59,1%)/9 (40,9%)	0.897
	Plasma concentration of atorvastatin and atorvastatin lactone combined			
	*Non-measurable (n=0)*	*Median or below (n=28)*	*Above median (n=29)*	*p-value*
Highest Ki67; median (iqr)	-	2.25 (1.425-3.925)	2.35 (1.725-3.725)	0.941
Median Ki67; median (iqr)	-	1.8 (1.2-3.225)	1.9 (1.1-2.575)	0.842
Intraprostatic inflammation score; median (iqr)	-	9 (8-12)	9 (8-11.75)	0.65
PSA baseline; median (iqr)	-	8.55 (5.525-14.1)	7.7 (5.2-10.25)	0.257
PSA before surgery; median (iqr)	-	8.2 (5.3-13.6)	6.2 (5-8)	0.065
PSA change; median (iqr)	-	-0.4 (-1.5-0.5)	-0.6 (-1.85-0.05)	0.658
3 ls score neg/pos; n (%)	-	13 (59,1%)/9 (40,9%)	12 (57,1%)/9 (42,9%)	0.897

Ki67 and PSA values, intraprostatic inflammation scores, and 3LS for placebo group are presented in Supplementary table 1.

### Follow-up analysis

During the follow-up period in the Pirkanmaa region, 26 patients (25.5%; 26/102) experienced biochemical recurrence, 7 died, and 6 patients had major acute cardiovascular events. Median time to biochemical recurrence following prostatectomy was 36 months (IQR 15-51). The pre-surgery statin intervention was not significantly associated with the risk of biochemical recurrence, with a hazard ratio (HR) of 1.54 (95% CI 0.68 to 3.45), and an adjusted HR (aHR) of 1.19 (95% CI 0.49 to 2.85). Additionally, no significant association was observed between statin intervention and overall mortality (aHR 1.07; 95% CI 0.24 to 4.90) or initiation of the hormonal treatment (aHR 2.19; 95% CI 0.41 to 11,67). However, the risk of MACE was lower in the atorvastatin arm (aHR 0.11; 95% CI 0.01 to 1.01) (Table 4).

**Table 4:** Endpoint events from the follow-up cohort (atorvastatin vs control) of patients with prostate cancer treated with radical prostatectomy and participating in ESTO1 clinical trial

	Univariable			Multivariable		
	HR	95% CI		aHR	95% CI	
Biochemical recurrence risk (n=26)	1,54	0,68	3,45	1,19	0,49	2,85
Biochemical recurrence risk (atorvastatin use 28 days or under) (n=13)	1,63	0,50	5,30	1,53	0,43	5,45
Biochemical recurrence risk (atorvastatin use over 28 days) (n=13)	1,46	0,48	4,46	1,21	0,31	4,74
MACE risk (myocardial infarct or stroke) (n=6)	0,14	0,02	1,19	0,11	0,01	1,01
Hormonal treatment risk (n=10)	1,73	0,45	6,70	2,19	0,41	11,67
Death risk (n=7)	1,02	0,23	4,54	1,07	0,24	4,90

In the full follow-up cohort, a total of 35 (22%) patients experienced biochemical recurrence. Median time to biochemical recurrence was 34 months (IQR 12–49). In this cohort, there were 10 deaths and 6 strokes. Atorvastatin treatment was not associated with biochemical recurrence (aHR 0.90; 95% CI 0.45 to 1.81). No significant association was observed between statin intervention and overall mortality (aHR 1.31; 95% CI 0.35 to 4.92), MACE (aHR 0.17; 95% CI 0.02 to 1.49), or initiation of hormonal treatment (aHR 1.32; 95% CI 0.41 to 4.24) (Table 5).

**Table 5:** Endpoint events from the full cohort (atorvastatin vs. control) of patients with prostate cancer treated with radical prostatectomy and participating in ESTO1 clinical trial

	Univariable			Multivariable		
	HR	95% CI		aHR	95% CI	
Biochemical recurrence risk (n=35)	1,14	0,58	2,23	0,90	0,45	1,81
Biochemical recurrence risk (atorvastatin use 28 days or under) (n=18)	1,15	0,45	2,97	0,90	0,34	2,39
Biochemical recurrence risk (atorvastatin use over 28 days) (n=17)	1,14	0,44	2,97	0,98	0,33	2,91
MACE risk (myocardial infarct or stroke) (n=6)	0,19	0,02	1,63	0,17	0,02	1,49
Hormonal treatment risk (n=12)	1,38	0,44	4,35	1,32	0,41	4,24
Death risk (n=10)	0,97	0,28	3,36	1,31	0,35	4,92

### 3LS presence in the tissue

Amont the 114 men who underwent surgery and had their lipidome profiling performed on their prostate tissue samples, 48 (42.1%) exhibited the distinct three-lipid signature (3LS). At baseline, patients with 3LS had slightly higher ISUP/Gleason scores, indicating more aggressive tumors, compared to those without the 3LS. However, other clinical characteristics were similar between the groups (Table 1). During follow-up, 11 (30.6%) patients with 3LS experienced biochemical recurrence, compared to 16 (37.2%) in the 3LS-absent group. There was a higher proportion of hormone therapy initiations in the 3LS group (9.5% vs. 4.8%), and overall mortality was also elevated (10.4% vs. 4.5%), although the absolute numbers of these events remained low. In the adjusted multivariable Cox regression model, the presence of the 3LS signature was not associated with the risk of biochemical recurrence (aHR 0.57; 95% CI 0.23 to 1.41; p=0.224) nor the risk of overall death (HR 2.23; 95% CI 0.46 to 10.8; p=0.321). In univariable Cox model with the 3LS formula calculated score as flexible continuous predictor, some lower values (corresponding to lower concentrations of the three lipids) were associated with improved biochemical recurrence-free survival. However, no association was observed for values exceeding the 3LS signature cutoff (Supplementary Fig. 1).

## Discussion

In our follow-up study of men with localized prostate cancer who underwent prostatectomy, we performed analysis of the long-term outcomes and inspected intraprostatic risk markers and their connection to the clinical characteristics and prognosis of PCa. Many of them were, to our knowledge, inspected for the first time.

Until recently, it was unclear whether statins could achieve effective concentrations within the prostate [25]. In this study, we found that the intraprostatic concentrations of atorvastatin seemed to influence the pathological characteristics of the tissue, with especially atorvastatin lactone showing significant correlation with Ki67 and PSA markers. The more lipophilic atorvastatin acid is likely to be more readily available in the prostate tissue. These findings suggest that the local anti-proliferative effects of statins observed in vitro could also be achieved in vivo. However, this study cannot exclude the possibility that systemic effects of statins contribute to the intraprostatic changes observed. Moreover, the concentrations of atorvastatin and its direct metabolites across different bodily environments are likely highly intercorrelated.

Unlike atorvastatin, its lactone form is not active inhibitor of the HMG-CoA reductase although it is more readily converted by CYP3A4 to metabolites that have pharmacologic activity equivalent to atorvastatin [33]. Additionally, several cytotoxic and anticancer effects distinct from the cholesterol pathway have been solely attributed to the lactone forms of statins [[34], [35], [36]]. Future studies should quantify statin lactone concentrations to better understand its potential mechanisms of action against cancer cells.

PSA is currently the only clinically utilized marker in the follow-up of PCa. A reduction in PSA can indicate decreased inflammation as it is a marker for cell damage. PSA also has its effects in the expression of androgen receptors (AR) and AR-signaling. The intraprostatic tumor Ki67 proliferation marker indicates the proliferation phases of the cells, thus, is used as a biomarker to estimate the cancer progression. A lower Ki67 level reflects reduced cancer cell division. The inflammation score reflects the intraprostatic inflammation as the name suggests. While these markers are not routinely used clinically, they offer valuable insights into PCa progression and treatment effects at the cellular level. Although this study did not demonstrate survival benefits associated with reductions in these markers, plausible pathways exist by which such reductions could improve outcomes in PCa.

The short-term atorvastatin intervention prior to prostatectomy did not result in significant differences in clinical PCa endpoints during long term follow-up of the original window-of-opportunity trial. Neither can we find evidence against the hypothesis that the two groups have equal outcomes with the current sample size. However, the atorvastatin group exhibited a lower risk of cardiovascular events. The limited duration of the original intervention warrants consideration, as this study is the first prospective investigation of long-term outcomes following statin intervention in PCa patients. Our findings align with previous epidemiological studies failing to show association of statin use and Pca recurrence after prostatectomy [37,38] contrary to association with benefit in more advanced cases [12] and those under ADT therapy [19]. Thus, as also suggested by Murtola and Siltari (2021) [39], future prospective studies should explore higher statin doses and longer exposure during later disease stages, focusing on survival as the primary endpoint. Additionally, the observed benefits of statins seem to emerge in patients receiving concurrent hormonal treatment, highlighting the importance of selecting the appropriate clinical context for atorvastatin use.

Previous studies have associated the plasma measured three-lipid signature with worse prognosis of local [40] and advanced PCa [26,27,41]. In this study, patients with a 3LS as defined from prostate tissue exhibited somewhat worse baseline clinical characteristics, including higher PSA, Gleason scores, and Ki67 levels, yet these differenced did not translate into worse prognoses during follow-up among. When the cutoff value for the 3LS signature was disregarded, certain values derived from the 3LS formula were associated with improved survival. This underscores the importance of adopting modelling approaches that are not constrained by arbitrary risk marker cutoffs, thereby enhancing both applicability and robustness. These findings are not entirely unexpected; one study demonstrated that serum and prostate shared only 39% of the assessed metabolites by a large panel [42] suggesting poor correlation between the two environments – possibly also in their lipid profiles. Consequently, the prostate lipidome may not be a reliable proxy for corresponding serum lipid levels.

Our study had several strengths. The follow-up cohort was derived from the ESTO1 trial, a randomized, placebo-controlled study design that allows for stronger causal inferences. Participant adherence with atorvastatin therapy was excellent. Moreover, this is the first study, to our knowledge, to investigate the three-lipid signature (3LS) score in prostate tissue samples and after prostatectomy.

There were limitations to this study. The study cohort was homogenic, consisting exclusively of Caucasian men, which may limit the generalizability of the results to other ethnic groups. The exposure to atorvastatin was relatively short. We lack information on the potential post-trial use of statins or other health-related behaviors among participants that may have influenced the outcomes. Additionally, prostatectomy has an excellent prognosis in local PCa, necessitating a longer follow-up period to capture a sufficient number of clinical endpoints for more definitive conclusions. Consequently, multiple competing comorbidities causing dropouts may also have been present during the follow-up period, potentially obscuring any associations between statins or 3LS and cancer outcomes. With respect to 3LS, elevated sphingolipids are also known risk factors for adverse cardiovascular outcomes. Additionally, the lipidome was assessed post-intervention, which may have caused shifts in lipid levels and introduced potential disrupting effects.

The unavailability of complete follow-up data for participants residing outside the Pirkanmaa region further constrained our analysis. We also analysed the data for the full cohort to obtain more statistical power. However, not all biochemical relapses are known for the group residing outside the Pirkanmaa region, so we acknowledged that this causes a bias towards the null. This should be considered in the interpretation of the results. All analyses were post-hoc in nature, and when stratifying the study population based on atorvastatin and lactone concentrations, the resulting small subgroup sizes led to increased uncertainty in the estimates.

Results suggest that the local effects of atorvastatin in the prostate may be achieved with relatively low tissue concentrations. It is yet to remain an open question, whether systemic or local statin therapy is more important to cause these changes and whether this will result in better prognosis in PCa. Survival benefits could be expected with longer exposure to atorvastatin.

## Conclusions

Atorvastatin lactone concentration correlated with improved pathological markers, in contrast to atorvastatin alone. The intraprostatic atorvastatin and intraprostatic atorvastatin lactone seem to influence the prostate tissue. The pre-surgery statin intervention did not result in significant observed differences in long term prostate-cancer outcomes. However, the atorvastatin resulted in a lower risk for major acute cardiovascular events, underscoring the broader clinical utility of statins in Pca patients. The three-lipid signature measured from prostate tissue lipidome does not appear to be prognostic marker in localized PCa. Further research is needed with larger study cohorts and extended follow-up periods.

## CRediT authorship contribution statement

**Eemil Lehtonen:** Writing – review & editing, Writing – original draft, Visualization, Methodology, Formal analysis, Data curation. **Maiju Vertanen:** Writing – review & editing, Writing – original draft, Visualization, Formal analysis, Data curation. **Heimo Syvälä:** Writing – review & editing, Validation, Methodology, Investigation. **Teemu Tolonen:** Writing – review & editing, Validation, Investigation, Data curation. **Seppo Auriola:** Writing – review & editing, Validation, Methodology, Data curation. **Teuvo Tammela:** Writing – review & editing, Project administration, Funding acquisition. **Aino Siltari:** Writing – review & editing, Writing – original draft, Supervision, Resources, Project administration, Methodology, Funding acquisition, Conceptualization. **Teemu Murtola:** Writing – review & editing, Writing – original draft, Supervision, Resources, Project administration, Funding acquisition, Conceptualization.

## Declaration of competing interest

The authors declare that they have no known competing financial interests or personal relationships that could have appeared to influence the work reported in this paper.

## Appendix Supplementary materials

Image, application 1
