# Supplementary material for: The Impact of Atorvastatin on Intraprostatic Biomarkers – Prognostic Value of 3LS-score – Follow-up of ESTO1-Trial
Source: Neoplasia. 2025 Feb 6;61:101132. doi: 10.1016/j.neo.2025.101132 (PMC11851191; doi:10.1016/j.neo.2025.101132)
Supplement: Supplementary file 1 [file mmc1.docx]

**Supplemental Online Content**

**Supplementary table 1.**  Comparisons between placebo group and groups of atorvastatin users with measurable concentrations of atorvastatin and atorvastatin lactone

**Supplementary figure 1.** Risk of biochemical recurrence survival in the follow-up cohort of PCa patients (n=91) from univariable Cox regression model, presented as a function of a smoothly modeled P-term.

This supplemental material has been provided by the authors to give readers additional information about their work.

**Supplementary figure 1.** Risk of biochemical recurrence survival in the follow-up cohort of PCa patients (n=91) from univariable Cox regression model, presented as a function of a smoothly modeled P-term using restricted cubic splines, indicates that for some values below P<0.5 there seems to be an association with lower risk (log-HR values above 0). However, for 𝑃≥0.5, the direction of the association remains unclear due to wide confidence intervals.

**
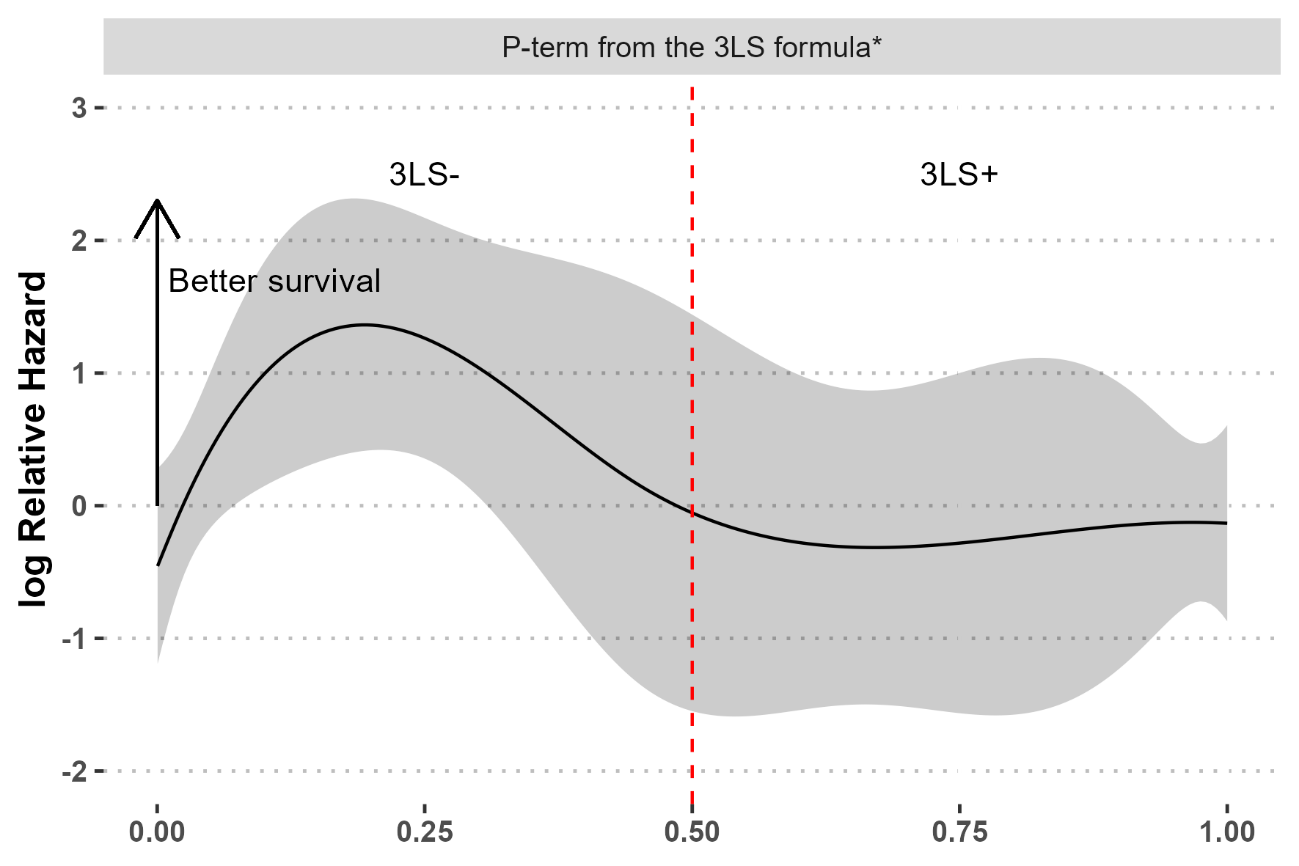
**

P is defined as P=e^y^/(1+e^y^), where y=3.1319*c1+2.1724*c2+1.8593*c3. Here, c1, c2, and c3 are the concentrations of ceramide, sphingomyelin, and phosphatidylcholine, respectively. Patients were considered to have the 3LS signature if P≥0.5.

**Supplementary table 1. Comparisons between placebo group and groups of atorvastatin users with measurable concentrations of atorvastatin and atorvastatin lactone**

|  | **Highest Ki67; median (iqr)** | **Median Ki67; median (iqr)** | **Intraprostatic inflammation score; median (iqr)** | **PSA baseline; median (iqr)** | **PSA before surgery; median (iqr)** | **PSA change; median (iqr)** | **3 ls score neg/pos; n (%)** |
| --- | --- | --- | --- | --- | --- | --- | --- |
| *Placebo (n=78)* | 2.5 (1.4-3.4) | 1.9 (1.1-2.5) | 9 (7-12) | 7.6 (5.35-10.85) | 7.55 (5.5-10.725) | -0.2 (-1.1-0.4) | 36 (46.2%))/24 (30.8%) |
| **Tissue concentration of atorvastatin** |  |  |  |  |  |  |  |
| *Non-measurable (n=15)* | 3.2 (1.7-4.4) | 2.5 (1.4-3.6) | 10 (9-12) | 5.6 (4.4-7.9) | 6.25 (4.1-7.975) | -0.4 (-1.225-1.125) | 4 (50%)/ 4 (50%) |
| *p-value, placebo* | 0.2 | 0.183 | 0.408 | 0.071 | 0.089 | 0.9 |  |
| *Measurable (n=42)* | 2.3 (1.25-3.55) | 1.8 (1.1-2.7) | 9 (8-12) | 8.7 (5.85-13.625) | 7.25 (5.45-12.875) | -0.65 (-2.175-0.15) | 21 (60%)/14 (40%) |
| *p-value, placebo* | 0.740 | 0.954 | 0.877 | 0.159 | 0.449 | 0.084 |  |
| *Median or below (n=14)* | 2.14 (1.175-3.625) | 1.2 (0.9-3.1) | 9.5 (8-12) | 8.7 (4.625-14.2) | 10.1 (5.2-12) | -0.450 (-1.375-0.425) | 5 (41.7%)/ 7 (58,3%) |
| *p-value, placebo* | 0.266 | 0.424 | 0.381 | 0.362 | 0.572 | 0.362 |  |
| *Above median (n=28)* | 2.3 (1.8-3.5) | 1.8 (1.275-2.625) | 8 (7-11) | 8.7 (6.15-13.475) | 9 (4.95-9.75) | -0.9 (-2.625-0.1) | 16 (69,6%)/ 7 (30,4%) |
| *p-value, placebo* | 0.762 | 0.659 | 0.707 | 0.219 | 0.539 | 0.1 |  |
| **Tissue concentration of atorvastatin lactone** |  |  |  |  |  |  |  |
| *Non-measurable (n=4)* | 3.8 (3.625-8.325) | 3.45 (2.7-5.775) | 9 (5-13.75) | 5.67 (3.11-13.375) | 7.5 (3.65-14.35) | 1.05 (0.15-2.145) | 2 (66,7%)/ 1 (33,3%) |
| *p-value, placebo* | 0.089 | 0.072 | 0.791 | 0.857 | 0.399 | 0.022 | 0.938 |
| *Measurable (n=53)* | 2.25 (1.425-3.65) | 1.75 (1.175-2.8) | 9 (8-12) | 8.4 (5.55-12.05) | 7.15 (5.1-10.95) | -0.6 (-1.975-0.075) | 23 (57,5%)/ 17 (42,5%) |
| *p-value, placebo* | 0.958 | 0.820 | 0.647 | 0.694 | 0.788 | 0.051 | 0.938 |
| *Median or below (n=25)* | 2.3 (1.4-3.85) | 1.95 (1.125-3.075) | 10 (8-12) | 5.8 (5-10.25) | 6.6 (5.3-9.45) | -0.5 (-1.3-(-0.1)) | 8 (47,1%)/ 9 (52,9%) |
| *p-value, placebo* | 0.918 | 0.576 | 0.392 | 0.264 | 0.131 | 0.165 | 0.692 |
| *Above median (n=28)* | 2.1 (1.4-3.5) | 1.75 (1.175-2.375) | 9 (8-11) | 9.25 (6.675-13.475) | 9.4 (4.95-10) | -1.25 (-2.625-0.25) | 15 (65,2%)/ 8 (34,8%) |
| *p-value, placebo* | 0.855 | 0.849 | 0.906 | 0.094 | 0.320 | 0.090 | 0.692 |
| **Plasma concentration of atorvastatin** |  |  |  |  |  |  |  |
| *Measurable (n=55)* | 2.3 (1.65-3.725) | 1.8 (1.2-2.875) | 9 (8-12) | 8.4 (5.5-11.6) | 7.2 (5.1-11.575) | -0.5 (-1.6-0.325) | 24 (43.6%)/18 (32.7%) |
| *p-value, placebo* | 0.793 | 0.579 | 0.625 | 0.730 | 0.917 | 0.145 | 0.773 |
| *Median or below (n=28)* | 2.25 (1.55-3.925) | 1.8 (1.2-3.225) | 9 (8-12) | 8.3 (5.525-14.1) | 8.2 (5.1-13.6) | -0.4 (-1.5-0.5) | 13 (59,1%)/ 9 (40,9%) |
| *p-value, placebo* | 0.784 | 0.583 | 0.670 | 0.505 | 0.425 | 0.461 | 0.925 |
| *Above median (n=29)* | 2.35 (1.325-3.725) | 1.9 (1.1-2.575) | 9 (8-12) | 8.4 (5.2-10.25) | 6.2 (5-8) | -0.7 (-1.85-0.05) | 12 (57,1%)/ 9 (42,9%) |
| *p-value, placebo* | 0.889 | 0.748 | 0.728 | 0.899 | 0.335 | 0.114 | 0.925 |
| **Plasma concentration of atorvastatin lactone** |  |  |  |  |  |  |  |
| *Measurable (n=55)* | 2.3 (1.65-3.725) | 1.8 (1.2-2.875) | 9 (8-12) | 8.4 (5.5-11.6) | 7.2 (5.1-11.575) | -0.5 (-1.6-0.325) | 24 (43.6%)/18 (32.7%) |
| *p-value, placebo* | 0.793 | 0.579 | 0.625 | 0.730 | 0.917 | 0.145 | 0.773 |
| *Median or below (n=28)* | 2.3 (1.425-3.925) | 2.1 (1.2-3.225) | 9 (7.25-12) | 9.65 (5.125-14.1) | 8.2 (5.1-13.6) | -0.4 (-2.4-0.5) | 12 (57,1%)/9 (42,9%) |
| *p-value, placebo* | 0.622 | 0.303 | 0.920 | 0.458 | 0.396 | 0.472 | 0.959 |
| *Above median (n=29)* | 2.3 (1.725-3.725) | 1.75 (1.075-2.575) | 10 (8-12) | 7.7 (5.5-9.95) | 6.8 (5-8) | -0.6 (-1.6-0.05) | 13 (59,1%)/9 (40,9%) |
| *p-value, placebo* | 0.940 | 0.859 | 0.499 | 0.855 | 0.331 | 0.114 | 0.959 |
| **Tissue concentration of atorvastatin and atorvastatin lactone combined** | | |  |  |  |  |  |
| *Non-measurable (n=2)* | 3.8 (3.7-.) | 3.05 (2.5-.) | 7 (4-.) | 5.67 (4.34-.) | 7.5 (6.8-.) | 1.83 (1.2-.) | 0 (0%)/1 (100%) |
| *p-value, placebo* | 0.139 | 0.14 | 0.43 | 0.211 | 0.973 | 0.044 | 0.491 |
| *Measurable (n=55)* | 2.3 (1.475-3.725) | 1.8 (1.2-2.875) | 9 (8-12) | 8.4 (5.5-12.5) | 7.15 (5.1-11.575) | -0.55 (-1.725-0.1) | 25 (59,5%)/17 (40,5%) |
| *p-value, placebo* | 0.905 | 0.65 | 0.625 | 0.666 | 0.594 | 0.042 | 0.491 |
| *Median or below (n=26)* | 2.25 (1.45-4.1) | 1.7 (1.15-3.3) | 10 (8-12) | 6.65 (4.85-10.7) | 6.2 (4-10.75) | -0.4 (-1.3-0.15) | 9 (50%)/9 (50%) |
| *p-value, placebo* | 0.787 | 0.551 | 0.307 | 0.477 | 0.265 | 0.295 | 0.459 |
| *Above median (n=29)* | 2.3 (1.5-3.475) | 1.8 (1.2-2.6) | 8.5 (7.25-11) | 8.7 (6.3-13.45) | 7.3 (5.75-11.75) | -1.1 (-2.55-0.1) | 16 (66,7%)/8 (33,3%) |
| *p-value, placebo* | 0.943 | 0.891 | 0.832 | 0.184 | 0.839 | 0.033 | 0.459 |
| **Plasma concentration of atorvastatin and atorvastatin lactone combined** | | |  |  |  |  |  |
| *Measurable (n= 57)* | 2.3 (1.55-3.775) | 1.8 (1.2-2.925) | 9 (8-12) | 8.4 (5.45-12.05) | 7.15 (5.1-10.95) | -0.5 (-1.6-0.25) | 25 (58,1%)/18 (41,9%) |
| *p-value, placebo* | 0.741 | 0.51 | 0.717 | 0.809 | 0.601 | 0.092 | 0.959 |
| *Median or below (n=28)* | 2.25 (1.425-3.925) | 1.8 (1.2-3.225) | 9 (8-12) | 8.55 (5.525-14.1) | 8.2 (5.3-13.6) | -0.4 (-1.5-0.5) | 13 (59,1%)/9 (40,9%) |
| *p-value, placebo* | 0.835 | 0.603 | 0.627 | 0.424 | 0.421 | 0.354 | 0.99 |
| *Above median (n=29)* | 2.35 (1.725-3.725) | 1.9 (1.1-2.575) | 9 (8-11.75) | 7.7 (5.2-10.25) | 6.2 (5-8) | -0.6 (-1.85-0.05) | 12 (57,1%)/9 (42,9%) |
| *p-value, placebo* | 0.75 | 0.595 | 0.926 | 0.689 | 0.112 | 0.08 | 0.99 |
